# Acceptability of the Cardiff Online Cognitive Assessment for Clinical Screening of Patients With Psychosis: Protocol for a Mixed Methods Study

**DOI:** 10.2196/84218

**Published:** 2026-03-03

**Authors:** Amy J Lynham, Anthony Cope, Sarah Milosevic, Ian R Jones, James T R Walters

**Affiliations:** 1 National Centre for Mental Health, Division of Psychological Medicine and Clinical Neurosciences, Cardiff University, Hadyn Ellis Building, Maindy Road, Cardiff, CF24 4HQ, United Kingdom, 44 02920688371; 2 Wales Suicide Prevention, Self Harm, Mental Health & Wellbeing Research and Evidence Network, NHS Wales, Cardiff, United Kingdom; 3 Centre for Trials Research, Cardiff University, Cardiff, United Kingdom; 4 Centre for Neuropsychiatric Genetics and Genomics, Division of Psychological Medicine and Clinical Neurosciences, Cardiff University, Cardiff, United Kingdom

**Keywords:** cognition, mental health, online, digital assessment, psychosis, clinical services, acceptability, implementation

## Abstract

**Background:**

The early detection of cognitive impairments in individuals with psychosis offers a means to support clinical and functional recovery. However, there are significant barriers to assessing cognition in clinical services, including lack of staff time, training, and confidence in administering assessments. We have developed the Cardiff Online Cognitive Assessment (CONCA), aiming to address these barriers, and here present the protocol to assess its acceptability as a clinical tool.

**Objective:**

The aim of this study is to conduct the early-stage testing of the CONCA as a clinical tool to determine whether it is acceptable to young people with a history of psychosis and to health professionals.

**Methods:**

This cross-sectional study will use a mixed methods approach. A total of 100 young people with a history of psychosis will complete the CONCA and an acceptability questionnaire. We will conduct qualitative interviews with a minimum of 20 participants with psychosis and 10 participants with professional experience of working in early intervention in psychosis services to explore opinions on the CONCA as a clinical tool, attitudes toward and barriers or facilitators of implementing the CONCA, and cognitive testing more generally in clinical services. Quantitative data will be analyzed using descriptive statistics and linear or logistic regression. Qualitative interviews will be analyzed using a deductive thematic analysis approach.

**Results:**

The enrollment of study participants started in July 2025 and is expected to end in October 2026. Data analysis is expected to be finalized by March 2027. As of September 15, 2025, we have enrolled 26 participants with psychosis in the quantitative arm of the study, and 4 participants with psychosis and 1 health professional in the qualitative arm.

**Conclusions:**

Our results will provide new data on the acceptability of the CONCA and cognitive testing more generally among patients and clinicians, as well as identify barriers and facilitators to the CONCA’s implementation. This will provide the groundwork for a larger hybrid effectiveness-implementation study.

## Introduction

### Background

Patients with psychosis experience significant cognitive impairments, up to 2.5 SDs below average, across a range of cognitive processes, including processing speed, working memory, verbal and visual memory, executive function, and social cognition [1,2]. These impairments impact community functioning, occupational functioning, social skills, and behavior to a greater extent than psychotic symptoms [3,4]. Cognitive problems are present early in the course of illness, have been identified before the onset of psychotic symptoms [5], and persist over time [6]. The early identification of these cognitive impairments enables early intervention to optimize clinical outcomes and support functional recovery.

Early intervention for young people experiencing psychosis is a global priority [7]. For example, in the United Kingdom, the National Institute for Health and Care Excellence recommends that all people presenting with their first episode of psychosis be referred to an early intervention in psychosis service (EIP) [8]. The remit of these services is to provide rapid assessment and tailored treatment for patients with psychosis to prevent relapse and promote recovery. The measurement of cognition as part of a comprehensive clinical assessment is recommended for patients with psychosis in national clinical guidelines across the world [8-11]. Cognitive assessment in EIPs could allow the early detection of cognitive problems and give clinical teams a broader understanding of their patients’ presentation, identify those at risk of poor outcomes, and allow for a more personalized approach to care [12]. Despite these potential benefits, formal cognitive assessments are not routinely undertaken due to constraints of staff time and training and the associated costs of available assessments due to licensing [13]. Studies have shown that patients have little insight into their own cognitive abilities, making the self-report of cognitive function unreliable, and thus more formal assessment is required [14,15].

There are numerous barriers to conducting cognitive assessments in clinical services for psychosis. Few cognitive assessments exist that were specifically designed for the clinical assessment of patients with psychosis, although there are several assessments that may be suitable candidates including the Brief Assessment of Cognition in Schizophrenia[16], Brief Cognitive Assessment[17], or the Brief Cognitive Assessment Tool for Schizophrenia[18]. In the United Kingdom, many of the cognitive assessments that are currently available to use in the National Health Service, such as the Montreal Cognitive Assessment, Mini-Mental State Examination, and Repeatable Battery for the Assessment of Neuropsychological Status, were designed to detect cognitive decline in older patients rather than the neurodevelopmental impairments associated with psychosis [11,19]. In addition, staff do not have sufficient time, training, or confidence to administer these tools and identify cognitive impairments in their patients [13,20,21]. Formal testing can be conducted by clinical psychologists, but given their responsibilities for psychological treatment, the time they have for cognitive assessment is minimal. Digital assessments with automated scoring may reduce the burden of cognitive testing for clinical staff. However, a systematic review of digital cognitive assessments for severe mental illness found that these assessments had (1) not been sufficiently validated, (2) had high licensing costs, (3) lacked normative data against which staff could benchmark performance, and (4) lacked data on acceptability among patients and clinical staff [22].

### Prior Work

The Cardiff Online Cognitive Assessment (CONCA) was developed as a brief web-based measure of cognition specifically for use in mental health research [23,24]. The CONCA addresses most of the barriers outlined above. It can provide a measure of overall cognition in as little as 10 minutes, so it is brief to administer. It can be completed on any internet-connected device and does not require a trained professional or specialist equipment to administer. The website was codeveloped with people with lived experience of psychosis and other severe mental illness and is simple to use. It has been validated against the gold standard pen and paper research assessment for psychosis, the MATRICS Consensus Cognitive Battery [23]. Performance on the CONCA is associated with the measures of disability [23]. Published normative data are available for the CONCA [24].

### Objectives

The CONCA is an established research tool, but less is known about its clinical utility. We have worked with health professionals from EIPs and people with lived experience to develop new features for the CONCA that would support its use in psychiatric services, providing personalized reports for patients and clinical teams to identify cognitive impairments and help them understand their cognitive functioning. However, the CONCA needs to be assessed for clinical acceptability before it can be implemented in services. The aim of this study is to determine whether the CONCA is acceptable to young people with a history of psychosis and health professionals and whether implementation in clinical services is feasible and to support the refinement of the CONCA and design of future testing and implementation. We will also evaluate attitudes toward cognitive assessment more generally. To do this, we will assess the acceptability and usability of the CONCA through questionnaires and qualitative interviews with participants with psychosis and health professionals.

## Methods

### Relevant Frameworks

#### Development of Digital Health Technologies

**Figure 1.**
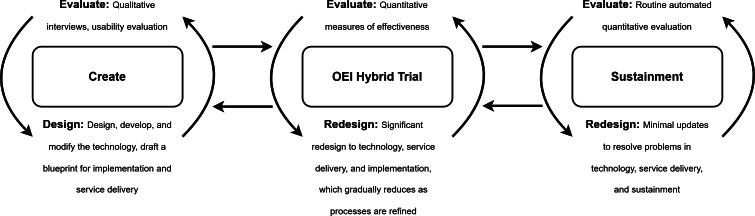
Accelerated creation-to-sustainment model adapted from Mohr et al [27], published under a CC BY 4.0 license [28]. OEI: Optimization, Effectiveness, and Implementation.

This project has been designed considering guidance from the National Institute for Health and Care Excellence Evidence Standards Framework for Digital Health and Care Technologies [25], the MRC Guidance for Developing and Evaluating Interventions [26], and Mohr et al’s [27] accelerated creation-to-sustainment model. The accelerated creation-to-sustainment model aims to accelerate the development of digital mental health technologies through an iterative process of design and evaluation across three phases: (1) Create; (2) Optimization, Effectiveness, and Implementation Hybrid Trial; and (3) Sustainment. Based on this model, the CONCA is currently in the *Create* phase, and the research outlined in this protocol will move the project to the next phase, *OEI Hybrid Trial* (Figure 1), by (1) developing a theory of how the CONCA will support services, (2) modeling outcomes, (3) testing procedures for evaluating the CONCA, and (4) providing data to support estimates of recruitment and retention for a larger study.

#### Technology Acceptance

Our measures of acceptability have been selected based on Sekhon et al’s [29] theoretical framework for evaluating the acceptability of health care interventions (theoretical framework of acceptability [TFA]). Sekhon et al [29] conceptualized acceptability into the following domains: affective attitude, burden, perceived effectiveness, ethicality, assessment coherence, opportunity costs, and self-efficacy. We will assess the acceptability of the CONCA tasks, the website design and functionality, user profile page and clinical interface, as well as attitudes toward cognitive testing as part of clinical care.

#### Consolidated Framework for Implementation Research

We will use the Consolidated Framework for Implementation Research (CFIR) [30,31] to support the identification of potential barriers and facilitators to the successful implementation of the CONCA in clinical services. The CFIR includes 5 constructs that might influence implementation, including the innovation itself, inner organizational settings, broader outer settings (such as the political or funding landscape), the individuals involved in implementation, and the process of implementation. We will use these constructs and the existing CFIR tools to develop our qualitative interview topic guide.

### Study Design

This cross-sectional study will use a mixed methods approach, where quantitative and qualitative data will be collected in parallel to address the primary research question (equal-status concurrent design [32]). Participants with psychosis will first be recruited into the quantitative arm and will be asked whether they would be interested in completing a longer follow-up interview (qualitative arm). In addition, we will invite health care workers with professional experience working in EIPs to complete the qualitative arm of the study. Consent will be received separately for the 2 arms of the study.

### Participants

We will recruit a representative sample of 100 young people with a history of psychosis. The demographic composition of patients in EIPs is documented in the National Clinical Audit of Psychosis, and these data will be used as a guide during recruitment to monitor the representativeness of our sample. We will also compare the demographics of the quantitative and qualitative arms throughout recruitment. We will use a range of approaches to achieve our recruitment target, including (1) working with EIPs in Wales and England to establish and implement ways of offering potential participants the opportunity to take part, (2) liaising with voluntary organizations to promote the research, (3) attending events and conferences to raise awareness of the research, (4) inviting individuals already enrolled in previous or ongoing research studies where participants have indicated their willingness to be approached about future studies, and (5) using communication outlets (social media, website, podcasts, and blogs) to inform the public about the research. To be eligible to take part in the study, individuals must meet the following criteria:

- Aged 16 to 40 years
- Have a diagnosis of psychosis (including schizophrenia-spectrum disorders, transient and acute psychosis, and psychosis secondary to a mood disorder)
- No deficits in sight that would prevent the potential participant from completing the cognitive tasks
- Currently well enough to take part, not currently in hospital or under the care of a crisis resolution and home treatment team

In addition, we will recruit a minimum of 10 participants with professional experience working in EIPs to complete qualitative interviews. Participants will be recruited from a range of disciplines, including psychiatrists, psychologists, community psychiatric nurses, and occupational therapists.

### Cardiff Online Cognitive Assessment

#### Overview

The CONCA is a web-based cognitive battery developed at Cardiff University, consisting of 5 tasks (Table 1). The first 3 tasks form the core battery, and the final 2 tasks are optional extras, which provide data on more complex aspects of cognitive function. The individual tasks were originally developed for web-based platforms by The Many Brains Project [33,34], a not-for-profit organization set up by Harvard University. The brief assessment can be completed in 10 minutes and produces a measure of general cognitive ability that is comparable to more intensive assessments and is associated with functional outcomes [24]. The full assessment (including the optional tasks) takes 30 minutes to complete. The assessment is hosted on a purpose-built website co-designed with people with lived experience of severe mental illness and health professionals working in EIPs.

**Table 1.** Cardiff Online Cognitive Assessment (CONCA) tasks.

Domain	Task	Administration time (min)	Description
Brief assessment (10 min)			
Speed of processing	Digit symbol coding	3	During the task, a key of 9 symbols and corresponding numbers is shown at the top of the screen. Participants select the number that corresponds to a target symbol (1, 2, or 3). The outcome measure is the number of correct responses in 90 seconds.
Working memory	Backward digit span	3	Participants are presented with a sequence of numbers and must recall them in the reverse order. The outcome measure is the maximum length of number sequence the participant can recall backward.
Premorbid or crystallized IQ	Vocabulary	3	Participants are shown a target word and asked to select which of 4 words is the closest in meaning to the target word. The outcome measure is total correct out of 20.
Optional extras (up to 20 min)			
Social cognition	Morphed emotion identification	3	Participants are presented with a face and must decide whether the face looks angry, fearful, happy, or disgusted. The outcome measure is the number of correct responses out of 60 faces.
Reasoning and problem solving	Matrix reasoning test	15	Participants must select the image that best completes a logical pattern. The outcome measure was the number of correct responses out of 35 trials.

We have used the CONCA as a research tool since 2017. In that time, we have established the validity of the CONCA against a gold standard, gathered feedback from participants, and collected data on over 5000 participants without any ethical issues (>1200 from the National Centre for Mental Health [23] and >3800 from HealthWise Wales [24]). The CONCA has been well tolerated based on high completion rates and positive feedback from participants.

We have recently developed 2 new features for the CONCA to support clinical administration: (1) an interface for clinicians to allow them to assess patients and support them to interpret their patients’ results and identify areas of cognitive difficulties and (2) feedback for both patients and research participants to inform them about their own cognitive ability.

#### Clinical Interface

**Figure 2.**
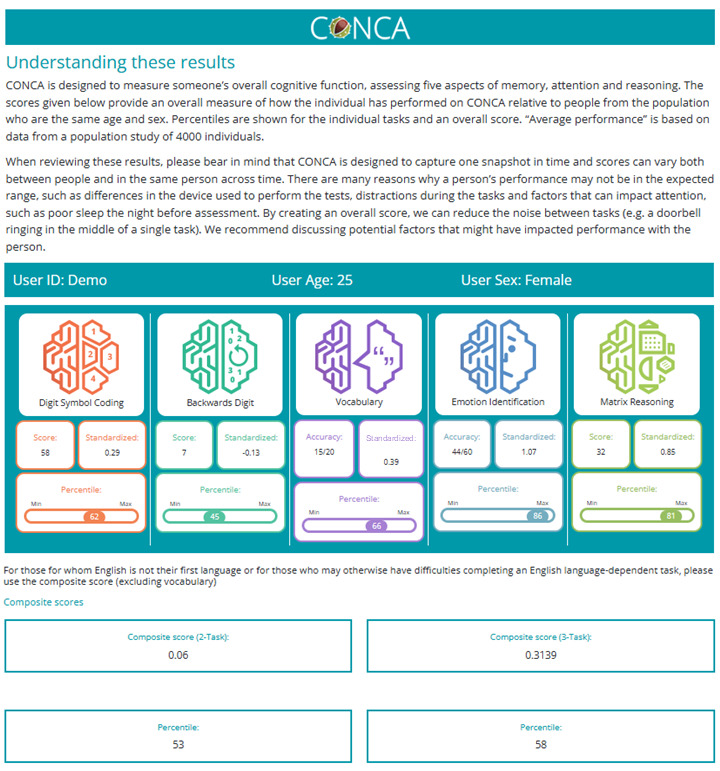
Screenshot from Cardiff Online Cognitive Assessment’s (CONCA) clinical interface. The results shown are dummy scores for illustrative purposes only.

The clinical interface is accessible for clinicians through an assigned login page where they can access and search for their patients’ cognitive results (patients are assigned a unique ID, so identifiable information is not required). Each cognitive report consists of (1) a guide to interpreting the results; (2) raw scores and age- and gender-corrected percentiles for each task; (3) an overall cognitive score; and (4) information on each task, what it measures, and how it relates to real-world functioning (Figure 2).

#### User Profile Page

**Figure 3.**
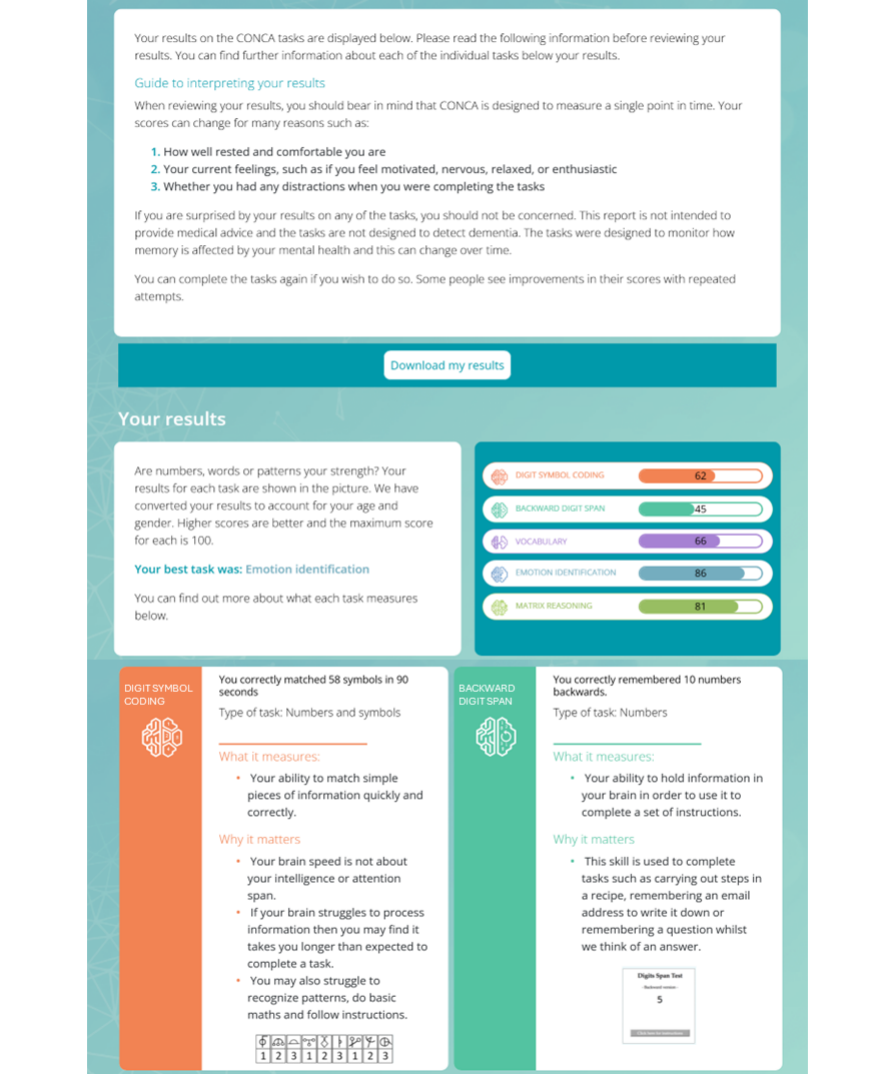
Screenshot of user profile page. The screenshot shows a section of the My Profile page as an example. Other features of the page are described above.

The user profile page differs from the clinical interface in that the focus is on individual performance rather than comparison with population data. Each profile page includes (1) a guide to interpreting the results; (2) raw scores on the tasks and information on what they mean; (3) graphical representation of the scores, highlighting the user’s best score; and (4) a graph tracking repeated attempts at each task so that users can see their performance over time (Figure 3).

### Data Collection

#### Quantitative Data Collection

Participants will complete the assessments on their own devices in an unsupervised setting. For participants who do not have access to the internet or their own device or would like support to complete the study, we will offer the option to complete the assessments on a study device either at home or at our research facility at Cardiff University.

Participants will first complete a brief set of questions on the web-based data collection tool, REDCap (Research Electronic Data Capture), including demographic information, current diagnosis, questions about the device they are using and their internet literacy, and subjective cognitive complaints (PROMIS v2.0 Cognitive Function–Short Form 4a [35]). On the completion of these questions, participants will be asked to complete all 5 cognitive tasks in the CONCA. The tasks will open in a separate browser tab on the CONCA website [36]. Their participant ID number will be entered automatically to allow us to link the data collected in REDCap with the data collected in the CONCA. Participants will also be instructed to review their results on their CONCA profile page.

On the completion of the CONCA, participants will be directed back to REDCap to complete a feedback questionnaire about the CONCA. We will use an adapted version of Sekhon et al’s [37] questionnaire to assess attitudes toward (1) the CONCA website, (2) the CONCA tasks, and (3) the feedback given on the participant profile page. We will also include questions about attitudes toward cognitive testing as part of clinical care. A full list of questions can be found in Multimedia Appendix 1. These questions were developed in collaboration with our public advisory group. Participants will be offered £20 (US $27) by bank transfer or gift voucher as a thank you for their time.

#### Qualitative Data Collection

For participants with psychosis, the purpose of the qualitative interviews will be to gather information on the acceptability of the CONCA and the profile page that provides feedback on scores, as well as the experiences of cognitive difficulties and attitudes toward cognitive testing in clinical services. For participants with professional experience working in EIPs, we will specifically focus on the clinical reports generated by the CONCA and the feasibility and acceptability of implementing the CONCA in EIPs.

Purposive sampling will be used to select participants for interview to ensure that respondents are as diverse as possible in respect of age, gender, and ethnicity. Participants with psychosis who completed the quantitative study above will be invited to complete a follow-up interview. We will utilize our existing links with EIPs across the United Kingdom to invite health professionals to the study. It is anticipated that our sample sizes will provide sufficient information power [38], as the study aim and sample population are highly specific. Pragmatic decisions on sample size will be made during the data collection process, informed by the assessments of data saturation for each identified theme.

Topic guides were developed with input from our public advisory group and guided by the themes included in the TFA and CFIR. We will adopt an iterative approach to developing our topic guides, refining the questions and prompts as the transcripts are analyzed and themes emerge. For participants with psychosis, interviews will follow a topic guide to address the following areas:

- Digital literacy and confidence
  - Confidence and familiarity using the internet generally
  - Confidence in navigating the CONCA website
- Design of the CONCA
  - Appearance
  - Format
  - Functionality
- Content of the CONCA
  - Was the information on the CONCA useful and clear?
  - What could be changed?
  - What was missing?
- Use of the CONCA
  - How might the information on the CONCA be used by the participant?
  - Concerns about using the CONCA or how the participant’s data may be used
- Knowledge of cognitive function
  - Understanding of cognition and different types of thinking skills
  - Experience of cognition in relation to mental health, including cognitive difficulties
- Monitoring cognition in mental health services
  - Experience discussing cognition (memory, concentration, etc) with their care team
  - Attitudes toward cognitive testing in clinics

For participants with professional experience working in EIPs, we will conduct “think-aloud” interviews as participants navigate the CONCA report. Think-aloud interviews capture the thought processes of participants as they use tools and have been used in a range of health intervention studies [39]. Interviews will follow a topic guide to address the following areas:

- Design of CONCA-generated clinical report
  - Appearance and layout
  - Format
  - Functionality
- Content of CONCA-generated clinical report
  - Was the information useful and clear?
  - What could be changed?
  - What was missing?
- Using the CONCA in EIPs
  - Current approaches to assessing cognitive function in EIPs
  - Cognitive testing as a priority in EIPs
  - How would the CONCA be used with patients?
  - Support and infrastructure needed to deliver the CONCA

On the completion of the interview, all participants will be offered £20 (US $27) as a bank transfer or gift voucher as a thank you for their time.

### Analysis

#### Quantitative Data Analysis

To measure protocol adherence and acceptability, we will calculate completion rates for each task and conduct descriptive statistics for the feedback questionnaire (proportions and percentages). Each item in the acceptability questionnaire (Multimedia Appendix 1) is rated on a 5-point Likert scale. Following consultation with our public advisory group, it was agreed that *no opinion* or *neutral* responses would be grouped with positive responses as the evidence of acceptability. We will report the proportion of positive and negative responses to each item in the acceptability questionnaire. Table 2 shows how each questionnaire item maps onto the TFA domains.

**Table 2.** Theoretical framework of acceptability (TFA) domains and data collected.

TFA domain	TFA survey questions	Examples of qualitative themes
Affective attitude	How comfortable did you feel navigating the CONCA^a^ website? Did you like or dislike completing the CONCA tasks? Did you like or dislike being able to view your results using the “My Profile” page on the CONCA?	Positive or negative attitudes toward the CONCA website, tasks, profile page, or clinical interface.
Ethicality	I have concerns about the CONCA being an online assessment. I have privacy concerns around completing the CONCA. The CONCA website is accessible for people with psychosis. I have concerns about the CONCA tasks. I have personal, moral, or ethical concerns about the CONCA giving feedback to people on their performance on the tasks.	Privacy concerns, barriers to accessing the CONCA, and safety concerns (eg, impact of the CONCA on symptom exacerbation).
Self-efficacy	How confident did you feel navigating the CONCA website? How confident did you feel completing the CONCA tasks? The information on the profile page was easy to understand.	Understanding of the information contained in the CONCA, confidence using the CONCA, and familiarity with cognitive testing.
Burden	How much effort did it take to complete the CONCA tasks? How much effort did it take to review your results on the “My Profile” page?	Amount of information that needs to be processed to use it, time taken to complete, and level of difficulty navigating the CONCA.
Effectiveness	The CONCA has improved my understanding of my cognition^b^.	How the CONCA has improved the understanding of cognition, how CONCA meets service need, relationship between CONCA and real-world experiences.
Coherence	It is clear to me how the CONCA tasks measure my cognition^b^.	Clarity of information provided (text and figures), utility of graphs or scores.
Opportunity costs^c^	I would like my cognition to be assessed as part of my care. I would be happy for my psychiatrist to see my scores on the CONCA tasks. My cognition is a priority for me.	Willingness to give up some appointment time for the CONCA, priority for cognitive testing or CONCA compared to other priorities.

a CONCA: Cardiff Online Cognitive Assessment.

b Participants are provided with a definition of cognition (“Thinking skills, including memory, concentration and problem-solving”).

c The suggested question in Sekhon et al’s [37] questionnaire was not well received by our public advisory group and did not fit well with the study design. Therefore, these were replaced with questions about whether cognition is a priority for participants as part of their clinical care.

We have developed a set of thresholds to define *acceptable* in consultation with our public advisory group and taking into consideration our existing data on CONCA completion rates and previous feedback [23]. Based on these discussions, we established a *traffic light* system:

- Green: Clear evidence of acceptability (≥80% positive responses)
- Amber: Evidence of acceptability but some aspects require refinement (60%‐79% positive responses)
- Red: Limited evidence of acceptability (<60% positive responses)

We will apply these thresholds to each of the items in the acceptability questionnaire and the overall completion rate of the CONCA battery.

We will conduct secondary analyses to examine factors that predict participants’ responses to the acceptability questionnaire, including CONCA scores, current mental state, and demographic characteristics (binary logistic regression). Finally, we will examine the correlation between participants’ ratings of their cognition and performance on each CONCA task.

#### Qualitative Interview Analysis

Interviews will be professionally transcribed, and transcripts will be analyzed using a deductive thematic analysis approach. A coding framework will be developed based on the interview topic guides through discussion among team members to identify the key themes and subthemes. A sample of the transcripts will be independently double-coded. Analysis will be supported by the computer-assisted qualitative analysis software NVivo 15 (Lumivero). Data will be analyzed thematically, with particular attention to both positive and negative appraisals of the CONCA, as well as the contextual factors influencing participants’ perceptions and the perceived barriers and facilitators to implementation.

Acceptability will be defined as the extent to which participants perceive the CONCA as useful, user-friendly, and aligned with their needs or the needs of the EIP. This will be assessed by identifying themes relevant to the TFA, including participants’ positive and negative feelings and attitudes toward the CONCA, the burdens and costs associated with using the CONCA, ethical beliefs around the CONCA, perceived benefits of the CONCA, and willingness to use the CONCA.

#### Triangulation

The data from the quantitative and qualitative arms will be analyzed independently and in parallel (equal status concurrent design [32]). The results will be integrated at the point of interpretation of the data. Thus, we will corroborate the findings from the 2 approaches to address the study questions.

### Public Involvement

We have embedded public participation involvement and engagement (PPIe) across the CONCA project lifecycle. This started with the co-design of the CONCA website with people with lived experience of severe mental illness and health professionals from an EIP. This included public contributions to the interface, logo, and graphics and the extensive testing and refinement of the website. The clinical features were also co-designed and tested by our PPIe group, resulting in substantial changes to ensure the feedback for patients and health professionals produced by CONCA was useful for these groups.

At the outset of the current project, we expanded our PPIe group to ensure a wide range of viewpoints from lived experience experts. The functions of this group are wide-ranging and include developing assessment materials, preparing patient consent forms and information sheets (ensuring lay language is used throughout), reviewing and selecting study questionnaires, and co-designing and reviewing updates to the CONCA website. The group will be part of the team developing future protocols and strategies for full implementation in clinical settings. It will also play a key role in disseminating the outputs from the project to a wider audience, including patients, carers, health care professionals, and policy makers.

We have developed a research partner role and appointed one of our most experienced PPIe members. The research partner acts as a PPIe group liaison, providing peer-to-peer support and ensuring group tasks stay on track and all members are kept fully informed and happy with their roles. Our research partner has been involved in a wide range of activities beyond those listed above, including providing training for new staff and contributing to manuscript writing and other forms of dissemination (including talks and podcasts).

We will assess the impact of our public involvement work using the Public Involvement in Research Impact Toolkit [40].

### Plans for Dissemination

The results of this study will be disseminated within the scientific community through conference presentations and publications in peer-reviewed journals. We will update our research participants on the progress of the study through blog posts, podcasts, social media posts, hosted events, and annual reports. We will work with our public advisory group and departmental communications team to explore new ways of disseminating our findings to the public, including the National Health Service, service user groups, and charities that serve people with lived experience of psychosis.

### Ethical Considerations

The study will be conducted according to the guidelines of the Declaration of Helsinki. The study was approved by Wales Research Ethics Committee 2 on May 20, 2025 (16/WA/0323). Interested individuals will receive an information sheet outlining the study protocol and be given 48 hours to review this prior to screening. Interested individuals will be screened and given the opportunity to ask questions. Informed consent will be received from all participants over the phone or via a video call with a trained researcher. Consent will be received separately for the quantitative and qualitative data collection. Consent will be recorded electronically through the study website hosted on REDCap. All data will be pseudonymized with participant IDs and will not include any identifiable data. All published data, including quotes from qualitative interviews, will be anonymized to ensure participant confidentiality. All participants will be reimbursed for their time at a rate of £20 (US $27) per hour via bank transfer or gift card.

## Results

Funding was obtained from Health and Care Research Wales (Welsh Government, grant number: AF 24 01), and the funding commenced on October 1, 2024. Participant recruitment started in July 2025. As of September 15, 2025, we have enrolled 26 participants with psychosis in the quantitative arm of the study, and 4 participants with psychosis and 1 health professional in the qualitative arm. Data collection is expected to be completed by October 2026.

## Discussion

### Anticipated Findings

The CONCA has the potential to improve service delivery for patients with psychosis by enabling timely, reliable, and clinically informative cognitive testing to take place in clinics to support the early detection of cognitive problems, treatment planning, and predictions of prognosis. It is our intention to provide the CONCA on a not-for-profit basis to clinical teams. However, it is currently unclear how cognitive testing would fit into the care pathway for individuals with psychosis. There is a need for data on the acceptability of the CONCA and cognitive testing more generally among patients and clinicians.

The findings from our research will be reviewed with our public advisory group and will be used to identify the aspects of the CONCA that could be refined to ensure that it is usable and accessible and provides the data that clinical teams need. We will base any decision on how to move forward with testing the CONCA on our traffic light system of acceptability, along with our identified themes from the qualitative analysis. Acceptability is a multidimensional construct, and we consider each domain to be essential to the future implementation of the CONCA. Therefore, we will assess all TFA domains individually and consider them to hold equal weight. In the case of any domain of acceptability being rated *red*, the further testing of the CONCA will not take place before both quantitative and qualitative feedback is reviewed, the identified issues addressed, and this domain of acceptability has been remeasured. For domains rated *amber*, we will refine the CONCA based on our findings prior to further testing. For domains rated *green*, we will not make any changes in this area and move forward with testing (assuming other aspects of acceptability have been addressed).

This study will provide the groundwork for a larger hybrid effectiveness-implementation study [41]. Working with our public involvement group and health professionals, we will review the data and themes that emerged from our qualitative analysis to identify facilitators and barriers to the CONCA’s implementation and reach a consensus on the changes to service delivery we hope to achieve and how we will measure these. We will develop a protocol that measures the outcomes and impacts of interest.

### Strengths and Limitations

A key strength of this study is the involvement of people with lived experience of psychosis and other severe mental illnesses. Our public members contributed to (1) the design of the CONCA at every stage; (2) the development of the study assessments, interview topic guides, information sheets, and consent forms; (3) our recruitment strategy; (4) the equality impact assessment for the study; and (5) the funding application process. They will also have opportunities to contribute to the analysis and dissemination of the study findings. We have applied several existing frameworks to ensure the robustness of our study design, including the CFIR, TFA, and regulatory frameworks for developing digital health technology. The use of these frameworks will ensure that we gather the evidence needed to support the future implementation and sustainability of the CONCA.

The use of a digital cognitive assessment risks excluding certain people, including those with low digital literacy, anxiety or paranoia around digital technology, lack of access to an internet-connected device, or existing cognitive impairment. We have put several mitigations in place to ensure that those who wish to take part can do so, regardless of technology access or familiarity. These include providing support to complete the study (in person or by phone) and providing a device if needed. However, there remains the possibility that people will decline to take part in the study due to the issues listed above. We will calculate completion rates as one aspect of acceptability. However, these may not accurately reflect completion in a clinical setting. Participants may be more likely to complete the tasks as part of this study, as they will have to answer questions about the tasks and will be given remuneration for their time. We will compare rates from this study with our existing data collected as part of our nonremunerated, unsupervised studies [23,24].

There is a risk of allegiance bias, as the CONCA is being evaluated by researchers who were involved in the development of the tool. To mitigate this, the qualitative interviews will be independently double coded by one of the authors (SM), a qualitative researcher who was not involved in the development of the CONCA. We have also prespecified the analyses we intend to undertake and outlined clear thresholds that we will use to define acceptability. Finally, we will ensure that negative feedback is clearly reported, including the proportions of negative responses to the acceptability questionnaire and negative appraisals of the CONCA that arise during the qualitative interviews.

Finally, our recruitment of health professionals may be skewed toward those who are receptive to or interested in the use of digital technology or cognitive assessments in their services. We will use purposive sampling to recruit participants across a range of clinical disciplines, seniority levels, and locations to capture diverse perspectives. Researchers will keep a reflective log during recruitment, which will help us monitor and adjust our strategies as needed.

## Supplementary material

10.2196/84218 Multimedia Appendix 1 Acceptability questionnaire developed for the study.

10.2196/84218 Peer Review Report 1 Peer-review report from the Health and Care Research Wales Advanced Fellowship Review Committee.

## Abbreviations

CFIR: Consolidated Framework for Implementation Research
CONCA: Cardiff Online Cognitive Assessment
EIP: early intervention in psychosis service
PPIe: public participation involvement and engagement
REDCap: Research Electronic Data Capture
TFA: theoretical framework of acceptability
