# Supplementary material for: Acceptability of the Cardiff Online Cognitive Assessment for Clinical Screening of Patients With Psychosis: Protocol for a Mixed Methods Study
Source: JMIR Res Protoc. 2026 Mar 3;15:e84218. doi: 10.2196/84218 (PMC12978975; doi:10.2196/84218)
Supplement: Multimedia Appendix 1 [file resprot-v15-e84218-s001.pdf]

Title: Acceptability of the Cardiff ONline Cognitive Assessment (CONCA), a web-based tool for clinical assessment of cognition in patients with psychosis: Protocol for a mixed methods study – Supplementary Material

Running Title: Acceptability of CONCA - Supplementary Material

Authors: Amy J Lynham[1], PhD, Anthony Cope[1,2], BSc, Sarah Milosevic[3], PhD, Ian R Jones[1], MRCPsych, PhD, James T R Walters[1,4], MRCPsych, PhD

Affiliations: [1]National Centre for Mental Health, Division of Psychological Medicine and Clinical Neurosciences, Cardiff University; [2]Wales Mental Health Research and Evidence Network, NHS Wales; [3]Centre for Trials Research, Cardiff University; [4]Centre for Neuropsychiatric Genetics and Genomics, Division of Psychological Medicine and Clinical Neurosciences, Cardiff University

Corresponding Author:

Amy J Lynham

National Centre for Mental Health, Division of Psychological Medicine and Clinical Neurosciences

Cardiff University

Hadyn Ellis Building

Maindy Road

Cardiff

CF24 4HQ

Email: lynhamaj@cardiff.ac.uk

Supplementary Material 1: Acceptability Questionnaire

The corresponding domain from Sekhon's Theoretical Framework for Acceptability (TFA) for each question is shown in square brackets.

CONCA as a website

How comfortable did you feel navigating the CONCA website? [Affective Attitude]

- ☐ Very uncomfortable
- ☐ Uncomfortable
- ☐ No opinion
- ☐ Comfortable
- ☐ Very comfortable

I have concerns about CONCA being an online assessment. [Ethicality]

- ☐ Strongly disagree
- ☐ Disagree
- ☐ No opinion
- ☐ Agree
- ☐ Strongly agree

I have privacy concerns around completing CONCA. [Ethicality]

- ☐ Strongly disagree
- ☐ Disagree
- ☐ No opinion
- ☐ Agree
- ☐ Strongly agree

The CONCA website is accessible for people with psychosis. [Ethicality]

- ☐ Strongly disagree
- ☐ Disagree
- ☐ No opinion
- ☐ Agree
- ☐ Strongly agree

How confident did you feel navigating the CONCA website? [Self-efficacy]

☐ Not at all confident
☐ Not very confident
☐ No opinion
☐ Confident
☐ Very confident

If you would like to provide any further comments about the CONCA website (for example, the layout, design, features or text) or elaborate on any of your answers, please write your comments here.

CONCA Tasks

Did you like or dislike completing the CONCA tasks? [Affective Attitude]

☐ Strongly dislike
☐ Dislike
☐ No opinion
☐ Like
☐ Strongly like

How much effort did it take to complete the CONCA tasks? [Burden]

☐ No effort at all
☐ A little effort
☐ No opinion
☐ A lot of effort
☐ Huge effort

I have concerns about the CONCA tasks. [Ethicality]

☐ Strongly disagree
☐ Disagree
☐ No opinion
☐ Agree
☐ Strongly agree

It is clear to me how the CONCA tasks measure my cognition (thinking skills, such as memory, concentration and problem solving). [Coherence]

☐ Strongly disagree
☐ Disagree
☐ No opinion
☐ Agree
☐ Strongly agree

How confident did you feel completing the CONCA tasks? [Self-efficacy]

☐ Not at all confident
☐ Not very confident
☐ No opinion
☐ Confident
☐ Very confident

If you would like to provide any further comments about the CONCA tasks or elaborate on any of your answers, please write your comments here.

<u>CONCA Profile Page</u>

This section asks about the profile page, which provides participants with their results on the CONCA tasks.

Did you like or dislike being able to view your results using the “My Profile” page on CONCA? [Affective Attitude]

☐ Strongly dislike
☐ Dislike
☐ No opinion
☐ Like
☐ Strongly like

How much effort did it take to review your results on the “My Profile” page? [Burden]

☐ No effort at all
☐ A little effort
☐ No opinion
☐ A lot of effort
☐ Huge effort

I have personal, moral or ethical concerns about the CONCA website giving feedback to people on their performance on the tasks. [Ethicality]

☐ Strongly disagree
☐ Disagree
☐ No opinion
☐ Agree
☐ Strongly agree

CONCA has improved my understanding of my cognition (thinking skills, including memory, concentration and problem-solving skills). [Effectiveness]

☐ Strongly disagree
☐ Disagree
☐ No opinion
☐ Agree
☐ Strongly agree

The information on the profile page was easy to understand. [Self-efficacy]

☐ Strongly disagree
☐ Disagree
☐ No opinion
☐ Agree
☐ Strongly agree

How useful was the information on your profile page to you?

☐ No use to me
☐ Not very useful
☐ No opinion
☐ Useful
☐ Very useful

If you would like to add additional comments about the "My Profile" page or elaborate on any of your answers, please provide your comments here.

Is there anything else you would like to say about your experience completing CONCA and viewing your results today?

Clinical Applications

My cognition (thinking skills) has been affected by my mental health.

- ☐ Strongly disagree
- ☐ Disagree
- ☐ No opinion
- ☐ Agree
- ☐ Strongly agree

I would like my cognition (thinking skills) to be assessed as part of my care.

- ☐ Strongly disagree
- ☐ Disagree
- ☐ No opinion
- ☐ Agree
- ☐ Strongly agree

I would be happy for my psychiatrist to see my scores on the CONCA tasks. (Your results will NOT be shared as part of this study)

- ☐ Strongly disagree
- ☐ Disagree
- ☐ No opinion
- ☐ Agree
- ☐ Strongly agree

I would find CONCA useful to discuss my cognition (thinking skills) with my psychiatrist.

- ☐ Strongly disagree
- ☐ Disagree
- ☐ No opinion
- ☐ Agree
- ☐ Strongly agree

My cognition (thinking skills) is a priority for me.

- ☐ Strongly disagree
- ☐ Disagree
- ☐ No opinion
- ☐ Agree
- ☐ Strongly agree
