# Supplementary material for: Acceptability of the Cardiff Online Cognitive Assessment for Clinical Screening of Patients With Psychosis: Protocol for a Mixed Methods Study
Source: JMIR Res Protoc. 2026 Mar 3;15:e84218. doi: 10.2196/84218 (PMC12978975; doi:10.2196/84218)
Supplement: Peer Review Report 1 [file resprot-v15-e84218-s002.pdf]

Applicant name:	Amy Lynham
Host organisation:	Cardiff University
Application reference number:	AF-24-01 (AL)
Project title:	Cardiff ONline Cognitive Assessment (CONCA): An online tool for clinical monitoring of cognition in patients with psychosis

Review 1:

1.1 Project (the research)
Review the strengths and weaknesses of the proposed research

a) Strengths

My view is that this is a compelling research proposal, with a number of strengths. The case for assessing (and responding to) cognitive impairments in people with psychosis is well-made, and the project benefits from being clearly located within a larger programme of research led by the applicant. This has commenced with the initial development of CONCA, driven by the applicant, and extends to an anticipated follow-on study once this fellowship has completed. The follow-on will aim to test CONCA in a larger intervention project and it helps, I think, that this growing programme of investigation is framed as a complex intervention. This fellowship application, specifically, is part of a feasibility testing phase.

Specifically:

CONCA is an accessible, web-based, assessment tool for use by people with psychosis, with the detail described in Table 1 in the main body of the application form. It is designed to generate personal reports for both service users and practitioners relating to cognitive impairments, thus informing future support and intervention. It has also been through a period of recent refinement, with the help of people with lived experience. CONCA has already been used by over 5000 people with experience of services, but the case advanced in the fellowship is that younger adults with psychosis have not yet had the opportunity to make use of the tool. This is a key group, for whom early support might make a real difference, and recruitment of a representative sample of this population will take place through the NCMH.

Three components are described:

1. A qualitative phase, in which ‘think aloud’ interviews take place with 20 service users and 10 practitioners as they work through the current iteration of CONCA. This will be followed by a quantitative phase, in which 100 young adults with experience of psychosis will complete CONCA assessments and then complete a series of evaluation and outcome measures. The aim here is to understand service users’ and practitioners’ perceptions of CONCA, service users’ inclination to use it as a self-assessment, experience of it in its current, web-based, form and more.

2. A component in which CONCA's properties will be tested, through a four-week follow-up self-assessment with the same 100 people as described in (1) above, in order to measure reliability. A healthy control group will also be recruited.
3. A final component in which CONCA will be refined in the light of the knowledge gained in (1) and (2), and a protocol and logic model developed for the planned follow-on project.

The qualitative methods to be used here are described in relatively light detail, but I appreciate the pressure on word counts in applications like this. I do not have the technical background to assess the detail of the quantitative methods which are planned particularly in (2) above. Overall, though, the individual steps of the project, plus the plan to use existing HCRW-funded resources via the NCMH, make good sense and the whole is clearly designed to make a difference in practice. There is also a clear public and patient involvement arm to the study, with opportunities identified in the study design for service users to inform key parts of the project. I think the plan of work is feasible, is well-supported by Cardiff University and has the chance to make a real contribution.

b) Weaknesses

Whilst I can see the value of viewing this project proposal through an implementation science prism, which is referred to in objective 1, I think more might have been said about how literature from within this field will inform the proposal. The Consolidated Framework for Implementation Research (CFIR) is mentioned: how, practically put, will this infuse the design of this project? What is this intended to add?

1.2 Questions or Comments for Applicants
Do you have any questions or comments that you would like the applicant to respond to which **may** be raised at the interview panel?

1. Arising from the above: can more be said about how implementation science ideas will be used to inform the design of this fellowship project? This is mentioned in the application but is left rather vague.

Review 2:

1.1 Project (the research)
Review the strengths and weaknesses of the proposed research

a) Strengths
Transitioning a research to tool for use in clinical monitoring provides a good grounded base for developing a fellowship alongside as there is already some evidence in existence of the pertinence of the measure. There is a strong likelihood of progression within the project given the foundational work that has already been undertaken.

Strong team with a driven and motivated individual

Inclusion of PPI within the development of the tool so far and the continued involvement of the group to ensure continued engagement.

Potential outputs of the project could prove to be an efficient effective tool for use in clinical monitoring of a difficult population.

b) Weaknesses

The aims and focus shift a little throughout the application – the main aspects appearing to be development of reliability statistics for the CONCA and provide evidence of acceptability amongst patients and health professionals. However, later on the need to establish the fit of CONCA into the clinical pathway is introduced – which is an important aspect but needs clarity on the scope of the outputs expected from this project as currently this is not a specific objective. Clarity on how this would be achieved should be included.

Potential lack of identified quantitative support for the applicant and the identified courses may not be the most relevant for the actual analysis required. Would like to see this aspect fully supported.

1.2 Questions or Comments for Applicants

Do you have any questions or comments that you would like the applicant to respond to which **may** be raised at the interview panel?

The power calculations for quantitative sections are unclear – is this powered over time or within/between group?

The data analysis section does not provide enough information on the proposed methods to ensure that this is appropriate and related to the sample size calculation conducted. Could you elaborate on the anticipated analysis?

Interested in the progression criteria indicated for a next phase trial – what would these look like?

Is a trial the most sensible next step and what would this be trialling?
